# Effect of a SARS-CoV-2 Protein Fragment on the Amyloidogenic Propensity of Human Islet Amyloid Polypeptide

**DOI:** 10.1021/acschemneuro.4c00473

**Published:** 2024-11-25

**Authors:** Marvin Bilog, Jennifer Cersosimo, Iliana Vigil, Ruel Z. B. Desamero, Adam A. Profit

**Affiliations:** † PhD Programs in Chemistry and Biochemistry, the Graduate Center of the City University of New York, New York, New York 10016, United States; ‡ Department of Chemistry, York College of the City University of New York, Jamaica, New York 11451, United States

**Keywords:** human islet amyloid polypeptide, SARS-CoV-2 peptides, aggregation, molecular dynamic simulation, docking, amyloid fibril formation, biophysical assays

## Abstract

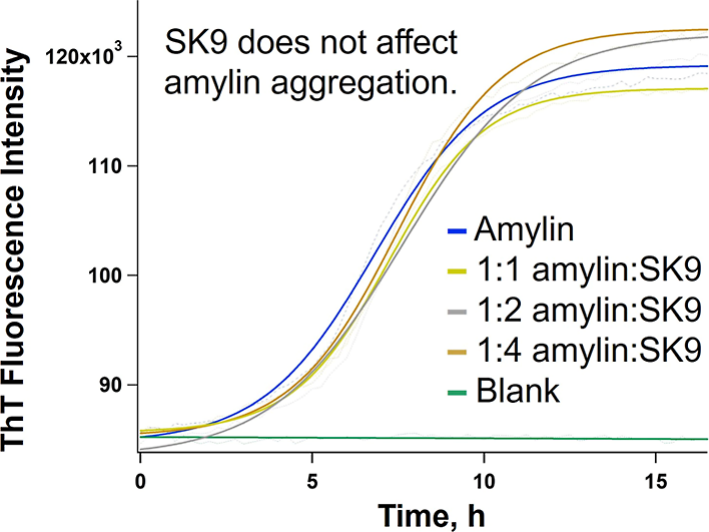

Infection with severe acute respiratory syndrome coronavirus 2 (SARS-CoV-2) and the onset of COVID-19 have been linked to an increased risk of developing type 2 diabetes. While a variety of mechanisms may ultimately be responsible for the onset of type 2 diabetes under these circumstances, one mechanism that has been postulated involves the increased aggregation of human islet amyloid polypeptide (hIAPP) through direct interaction with SARS-CoV-2 viral proteins. Previous computational studies investigating this possibility revealed that a nine-residue peptide fragment known as SK9 (SFYVYSRVK) from the SARS-CoV-2 envelope protein can stabilize the native conformation of hIAPP_1–37_ by interacting with the N-terminal region of amylin. One of the areas particularly stabilized through this interaction encompasses residues 15–28 of amylin. Given these findings, we investigated whether SK9 could interact with short amyloidogenic sequences derived from this region of amylin. Here, we employ docking studies, molecular dynamics simulations, and biophysical techniques to provide theoretical as well as direct experimental evidence that SK9 can interact with hIAPP_12–18_ and hIAPP_20–29_ peptides. Furthermore, we demonstrate that SK9 not only can interact with these sequences but also serves to prevent the self-assembly of these amyloidogenic peptides. In striking contrast, we also show that SK9 has little effect on the amyloidogenic propensity of full-length amylin. These findings are contrary to previous published simulations involving SK9 and hIAPP_1–37_. Such observations may assist in clarifying potential mechanisms of the SARS-CoV-2 interaction with hIAPP and its relevance to the onset of type 2 diabetes in the setting of COVID-19.

## Introduction

Human islet amyloid polypeptide (hIAPP), also known as amylin, is a 37-residue hormone stored and cosecreted with insulin by pancreatic β cells.^1^ The polypeptide plays an important role in the maintenance of glucose homeostasis and appetite satiation.^2^ Amyloid deposits composed of hIAPP_1–37_ are a hallmark of individuals afflicted with adult onset type 2 diabetes.^3,4^ Amyloids of hIAPP have long been thought to be responsible for the destruction of β cells and the corresponding loss of insulin production. However, growing evidence suggests that mature amyloid fibrils may not be pathological entities but rather they are soluble oligomers along the misfolding pathway that are the actual culprits of cellular toxicity.^5−8^ These oligomers are believed to disrupt the membranes of β cells leading to their death and depletion.^9−11^ Mechanisms of cellular toxicity include loss of ion homeostasis and proteolytic dysregulation as well as activation of death receptors and various apoptotic signaling pathways.^8,12^

Infection with severe acute respiratory syndrome coronavirus 2 (SARS-CoV-2), the causative agent of COVID-19, has been documented to induce diabetogenic metabolic changes and the onset of adult type 2 diabetes.^13−18^ Furthermore, individuals with pre-existing type 2 diabetes are more susceptible to SARS-CoV-2 infection. These individuals are at higher risk for severe disease, complications, and poor outcomes.^19^ Based on these observations, it has been postulated that SARS-CoV-2 infection may initiate type 2 diabetes through the interaction of viral proteins with hIAPP_1–37_. This interaction may enhance the amyloidogenic propensity of amylin, causing it to more readily self-assemble.

To test this hypothesis, Chesney et al. conducted molecular dynamics (MD) simulations to determine if SARS-CoV-2 surface proteins could affect the aggregation of hIAPP_1–37_.^20^ Simulations using a nonapeptide, designated SK9, encompassing residues 55–63 (SFYVYSRVK) of the C-terminal viral envelope protein were investigated. MD simulations with SK9 led to an amylin monomer structure with an increased helical content. Specifically, residues 15–28 were found to adopt an extended helical conformation at the end of the simulations. This extended helix was not observed in the control simulations of amylin without SK9. Furthermore, this extended helix differs from the helix-kink-helix conformation encompassing residues 15–28 previous reported for amylin.^21^ Based on these findings, Chesney et al. concluded that SK9 stabilizes amylin monomers.^20^ The added stability induced by SK9 should make amylin less prone to misfolding and forming amyloid. These results are markedly different from similar simulations determining the effect of SK9 on serum amyloid A and α synuclein, where SK9 was found to increase aggregation propensity.^22,23^

Chesney and co-workers also investigated the effects of SK9 on mature amyloid fibrils.^20^ MD simulations found that the viral peptide was capable of stabilizing fibrils. These investigators suggest that, by stabilizing fibrils, SK9 could drive the equilibrium from soluble monomers to mature fibrils, thereby increasing the yield of amyloid. Higher levels of amyloid could potentially exacerbate the symptoms of type 2 diabetes. Thus, a potential mechanism exists by which SARS-CoV-2 may worsen or initiate type 2 diabetes. These findings may assist in explaining why type 2 diabetes is a major comorbidity of COVID-19.

We were intrigued by the implication that SK9 could stabilize residues 15–28 of hIAPP_1–37_. This region encompasses portions of two highly amyloidogenic sequences of amylin. Both hIAPP_12–18_ (LANFLVH) and hIAPP_20–29_ (SNNFGAILSS) are well-characterized peptides with the 20–29 fragment, and associated derivatives, being extensively used as a model system for amyloid formation.^24−29^ Consequently, we wondered whether SK9 could interact with these two short amylin peptides. Here, we present findings from MD simulations as well as experimental evidence that SK9 can influence the amyloidogenic propensity of hIAPP_12–18_ and hIAPP_20–29_. In striking contrast, we also demonstrate that SK9 has little effect on the self-assembly of full-length amylin.

## Results and Discussion

**Figure 1:**
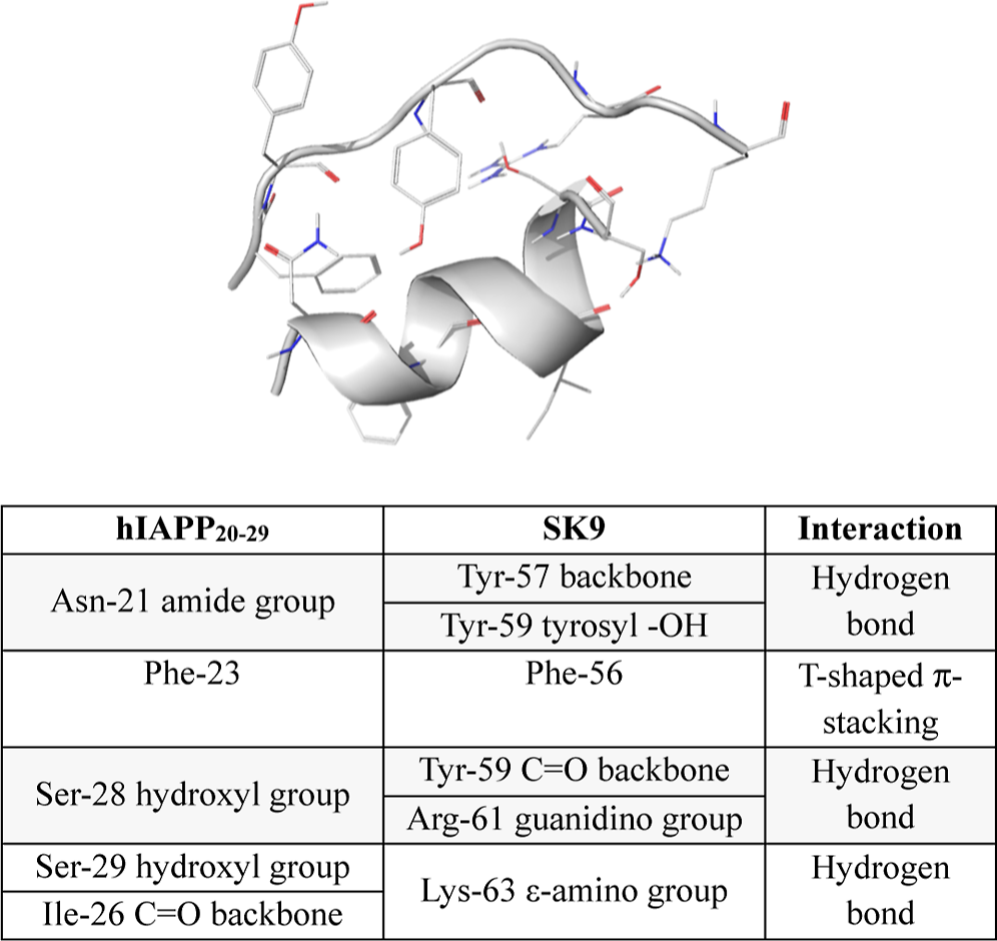
Configuration of the hIAPP_20–29_–SK9 system obtained after docking. Interacting residues are shown using sticks. The table provides a summary of specific interactions between SK9 and hIAPP_20–29_.

Previous computational studies predicted that SK9 stabilizes the native conformation of hIAPP_1–37_ and prevents its misfolding.^20^ As a consequence of this stabilization, residues 15–28 of hIAPP were found to exist as an extended helix, which differs from the initial helix-kink-helix structure.^21^ In addition, SK9 appeared to interact with the N-terminal region of amylin. This interaction seemed to propagate stability to the 15–28 region. Since residues 15–28 were stabilized in these simulations, we asked if SK9 could interact with short sequences from this region such as hIAPP_12–18_ and hIAPP_20–29_. Both sequences are well-studied model peptides encompassing amyloidogenic fragments of hIAPP that are capable of self-assembly. In particular, hIAPP_20–29_ and its variants have been used as experimental surrogates for full-length amylin. First, we used MD simulations to investigate the effect of SK9 on the structural stability of hIAPP_20–29_. We acquired the initial configuration of hIAPP_20–29_ from the NMR-resolved structure of hIAPP (PDB ID: 2L86)^21^ by removing residues 1–19 and 30–37. We docked hIAPP_20–29_ and SK9 in a 1:1 ratio using HADDOCK^30,31^ and used the configuration with the best score as input for the MD simulations (Figure 1). Simulations were run using the GROMACS 2022 package^32^ with the CHARMM 36m all-atom force field.^33^ Simulations were run for 1000 ns.

**Figure 2:**
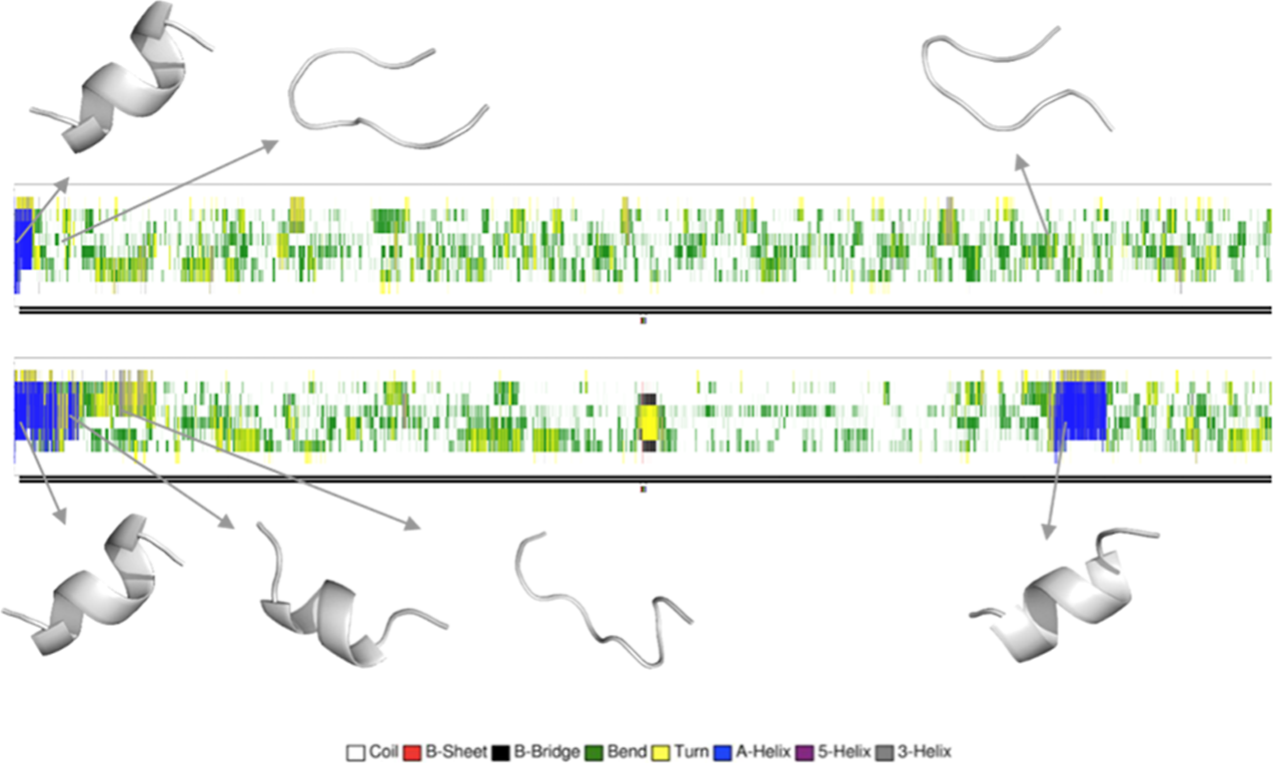
Secondary structure calculated by DSSP for each residue of hIAPP_20–29_ alone (top) and in the presence of SK9 (bottom) during the 1000 ns simulations. Also shown are the conformations of the peptides at different time frames. In the presence of SK9, the initial helical structure of hIAPP_20–29_ persisted longer and was regained after 820 ns.

The results of our MD simulations on hIAPP_20–29_ in the absence and presence of SK9 are shown in Figure 2. Simulations revealed that in the case of hIAPP_20–29_ alone, the peptide lost its helical structure after 25 ns and began to adopt a hairpin-like structure. The peptide continued to sample variations of this hairpin until the end of the simulation. In contrast, in the presence of SK9, the helical conformation of hIAPP_20–29_ persisted for over 25 ns. It took 102 ns before the peptide completely lost its helical conformation; this conformation was reestablished after 820 ns. Significantly, the root mean square deviation (rmsd) of the helical structure at 820 ns displayed only minor deviations from that of the initial helix, thereby indicating the two structures are very similar in nature. The results of these simulations support our hypothesis that SK9 can interact with hIAPP_20–29_. These findings are notably different from the MD simulations of Chesney et al. using full-length hIAPP_1–37_.^20^ In those computations, the final structure of the hIAPP-SK9 complex revealed that SK9 interacts more with the N-terminal region of hIAPP_1–37_, with residues 15–28 being stabilized in an extended α helix. There did not appear to be any binding between SK9 and the 15–28 region. Our data provides the first evidence that SK9 may be able to interact with hIAPP_20–29_.

**Figure 3:**
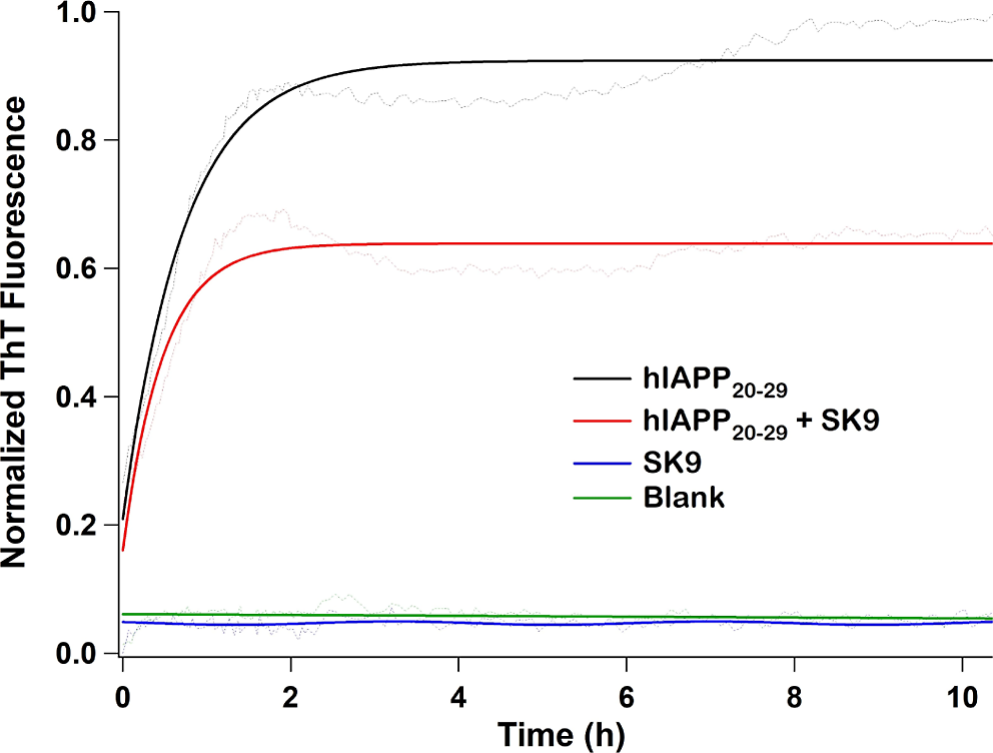
Time course ThT data for the self-assembly of hIAPP_20–29_ alone and in the presence of SK9. Data were obtained at room temperature using samples with a final concentration of 200 μM in 10 mM Tris-HCl buffer (pH 7.4) placed in a 96-well plate and run on a plate reader set to measure ThT fluorescence. Samples were excited at 450 nm, and their ThT fluorescence emission intensities were monitored at 486 nm. Measurements were taken every 10 min for at least 10 h.

To experimentally test the validity of our computational model, we investigated the ability of SK9 to inhibit amyloid formation by hIAPP_20–29_ using thioflavin T (ThT) kinetic aggregation assays. Figure 3 displays the results of these assays in the absence and presence of SK9. Clearly, SK9 inhibits the self-assembly of hIAPP_20–29_. These data corroborate our computational model and the hypothesis that the short hIAPP_20–29_ sequence can interact with SK9.

**Figure 4:**
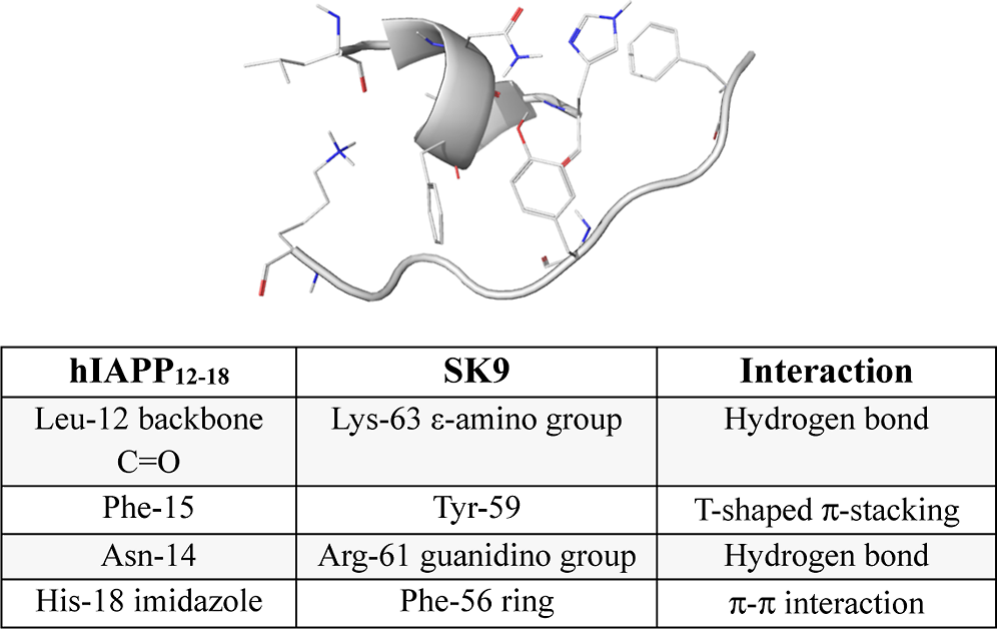
Configuration of the hIAPP_12–18_–SK9 system obtained after docking. Interacting residues are shown using sticks. Below is a table summarizing the observed interactions.

**Figure 5:**
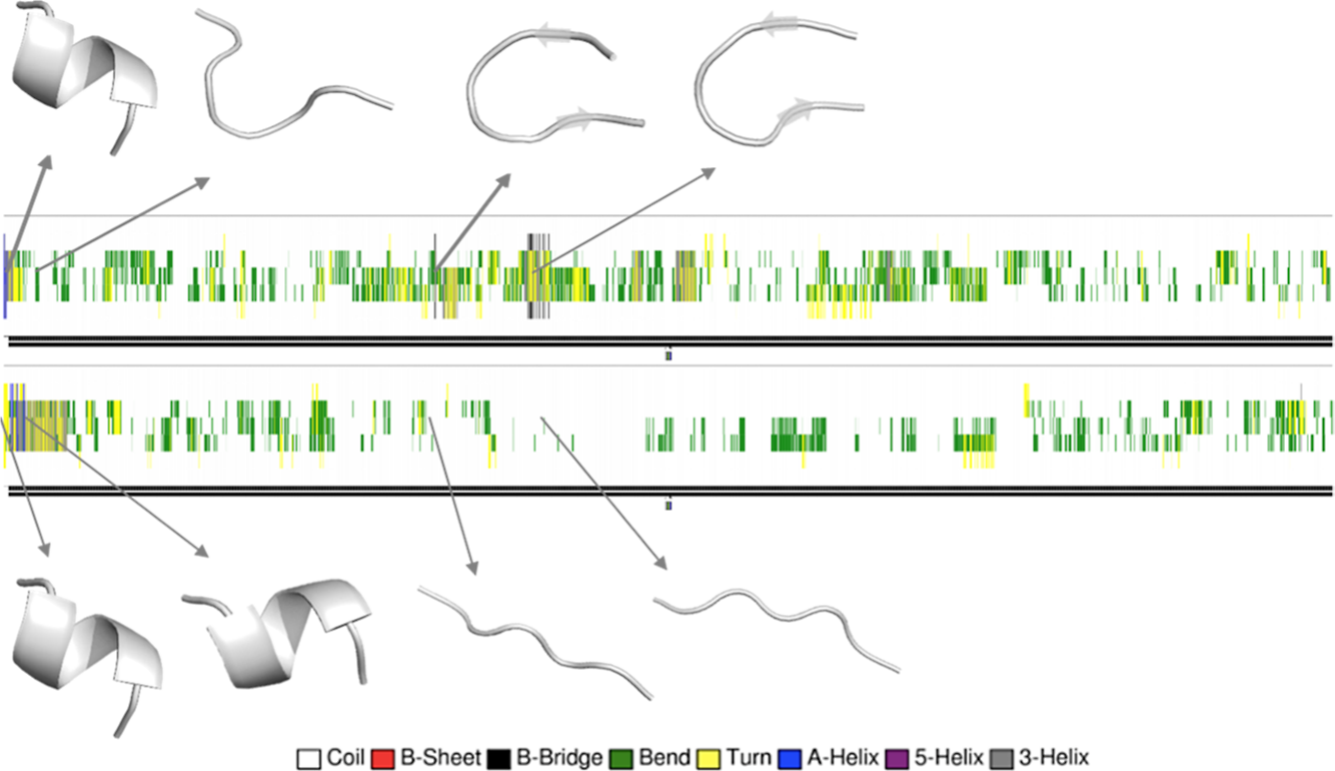
Secondary structure calculated by DSSP for each residue of hIAPP_12–18_ alone (top) and in the presence of SK9 (bottom) during the 1000 ns simulations. Also shown are the conformations of the peptides at different time frames. When SK9 was present, the helical conformation of hIAPP_12–18_ persisted longer, and the peptide remained disordered most of the simulation time. Without SK9, it took only a few nanoseconds before helical hIAPP_12–18_ devolved to random coils. The peptide also formed hairpin-like structures stabilized by a β bridge between Ala-13 and Val-17 at around 300–400 ns.

Next, we turned our attention to the hIAPP_12–18_ sequence to determine whether it could productively interact with SK9. Docking and MD simulations were conducted as previously described. Figure 4 displays the docked hIAPP_12–18_-SK9 structure along with a summary of key interactions. Highlights from the simulations are illustrated in Figure 5. In the absence of SK9, hIAPP_12–18_ lost its helical conformation after just 2.3 ns. In the presence of SK9, helical conformers persisted until 17.6 ns, when hIAPP_12–18_ shifted to random coils for the duration of the simulation. These findings again support the idea that SK9 can interact with hIAPP_12–18_.

**Figure 6:**
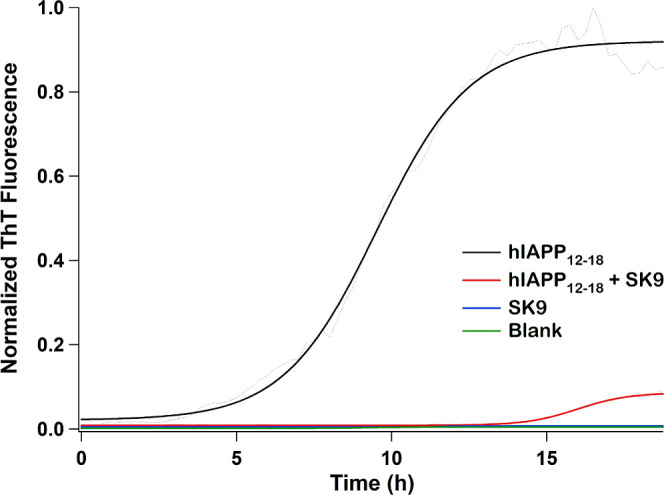
Time course ThT data for the self-assembly of hIAPP_12–18_ alone and in the presence of SK9. Data were obtained at room temperature using samples with a final concentration of 200 μM in 10 mM Tris-HCl buffer (pH 7.4) placed in a 96-well plate and run on a plate reader set to measure ThT fluorescence. Samples were excited at 450 nm, and their ThT fluorescence emission intensities were monitored at 486 nm. Measurements were taken every 15 min for at least 18 h.

To confirm the findings of MD studies, the propensity of hIAPP_12–18_ to form amyloid was monitored in the presence of SK9 by using ThT fluorescence assays (Figure 6). Evidently, SK9 is a potent inhibitor of hIAPP_12–18_ aggregation. SK9 virtually eliminates hIAPP_12–18_ self-assembly, with over a 90% reduction in amyloid. These data imply that hIAPP_12–18_ is a much better binding partner for SK9 than hIAPP_20–29_.

From the ThT data, it is also evident that SK9 is not capable of self-assembly within the time frame of the experiments (Figures 3 and 6). In a separate experiment, we monitored the ThT fluorescence spectra of SK9 for 16 days and observed no significant changes in the spectra. These findings demonstrate that SK9 does not form aggregates even during an extended incubation time (Figure S1). This observation is in stark contrast to the highly homologous TK9 peptide derived from the SARS-CoV envelope protein.^34^ TK9 (TVYVYSRVK) has been shown to form aggregates. The fact that SK9 (SFYVYSRVK) fails to aggregate confirms the importance of the VYVY motif in the self-assembly of TK9. The two Tyr residues in TK9 play a major role in the peptide’s ability to self-assemble through stabilizing π-stacking interactions between monomers. These aromatic–aromatic interactions occur between Tyr-3-Tyr-3, Tyr-5-Tyr-5, and Tyr-3-Tyr-5. Moreover, Val-2 appears to be critical in the aggregation of TK9 especially since it forms multiple hydrophobic interactions with Tyr-3, Val-4, and Tyr-5 in the aggregated form of TK9. Indeed, the deletion of Val-2 resulted in a significantly lower degree of aggregation of TK9 according to the same study. Computational data suggest the main stabilizing factors in the assembly of TK9 were the van der Waals interactions between TK9 monomers involving valine and tyrosine residues.^34^ Replacement of Val-2 with phenylalanine, as in the case of SK9, seems to have disrupted the ability of residue 2 to form the contacts necessary for self-assembly. Phe is more hydrophobic than Val with each residue having a hydrophobicity parameter of 1.79 and 1.22, respectively, as determined by their partition coefficients at the octanol/water interface compared to that of glycine.^35^ Therefore, a decrease in the level of hydrophobic interactions is not likely responsible for the failure of SK9 to aggregate. However, Val has a higher β-sheet propensity than Phe^36^ and this reduced inclination toward forming a β-sheet secondary structure along with the increased steric bulk of Phe may be the major contributing factors that prevent the self-assembly of SK9.

**Figure 7:**
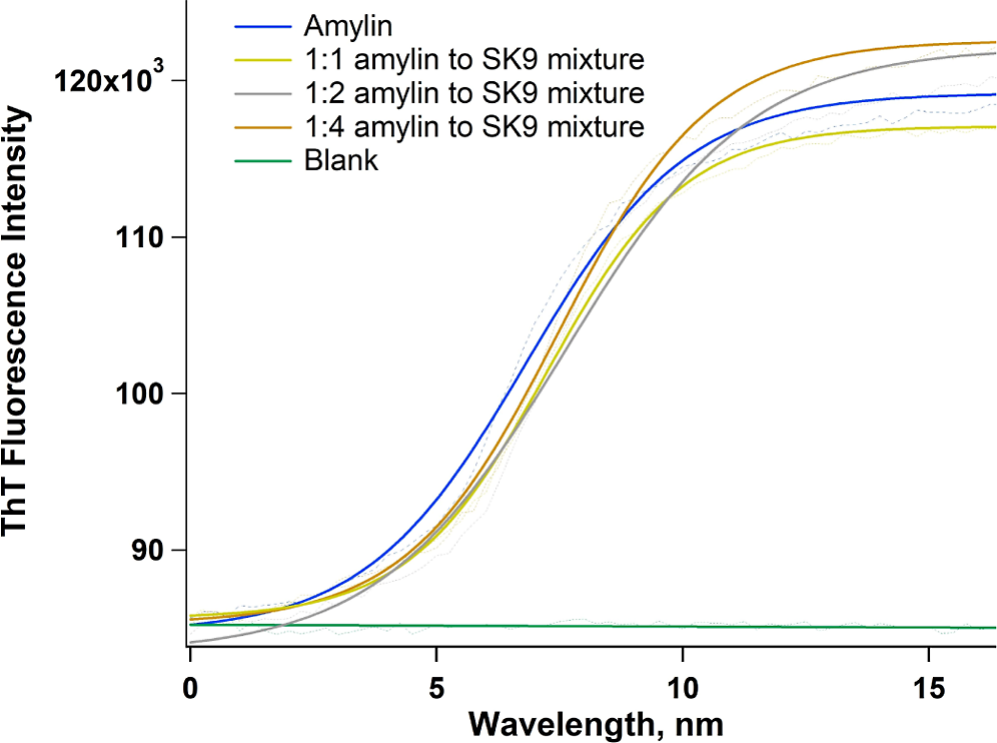
Representative ThT kinetic aggregation assay data illustrating the effect of SK9 on the amyloidogenic propensity of full-length amylin (hIAPP_1–37_). Aliquots of amylin (20 μM) were incubated with 0, 20, 40, and 80 μM SK9 and ThT fluorescence monitored as a function of time. SK9 was found to have no significant effect on amylin self-assembly kinetics.

Based on the ability of SK9 to interact with the hIAPP_12–18_ and hIAPP_20–29_ sequences, we set out to determine what effect it might have on the self-assembly of full-length amylin. MD studies from Chesney et al. imply that SK9 stabilizes hIAPP_1–37_ and impedes its aggregation.^20^ These simulations are consistent with previous experiments demonstrating that the related TK9 peptide inhibits amylin aggregation.^34^ To assess the ability of SK9 to interact with hIAPP_1–37_, we incubated the viral peptide with amylin at various concentrations and monitored the ThT fluorescence at different incubation times. Figure 7 shows the results of these studies. The data revealed that the amylin control aggregates as expected displaying typical ThT sigmoidal kinetics corresponding to lag, growth, and saturation phases. When 20 μM amylin was incubated with increasing concentrations of SK9, ranging from 20 to 80 μM, no major change in self-assembly kinetics relative to the control was observed. These findings demonstrate that SK9 does not significantly interact with hIAPP_1–37_ and is incapable of inhibiting amylin aggregation. These observations are in opposition to previous MD studies with SK9 and experimental evidence obtained using TK9.^20,34^ The results are also in stark contrast to our findings that SK9 can interact with short peptide fragments derived from the hIAPP_15–28_ region.

**Figure 8:**
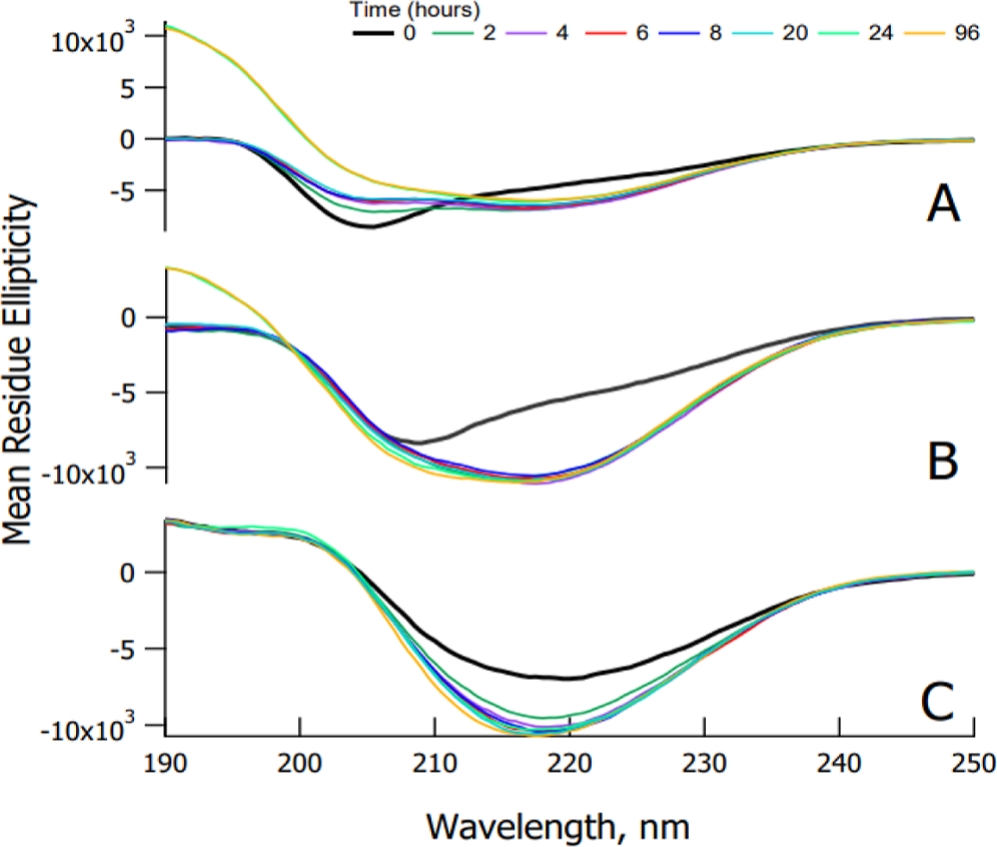
Time course of amylin self-assembly (A), amylin incubated with SK9 1:1 (B), and amylin incubated with SK9 1:4 (C) as monitored by CD. The changes occurring for amylin (A) over a 24 h period display a secondary structure shift from partial α helix (negative band at 205 nm at the 0 h trend) to β sheet (positive band at 196 nm and negative band at 218 nm at the 24 and 96 h trend). Amylin plus SK9 at 8 μM (B) shows slight structural changes during the first 3 h of incubation, but thereafter the spectra show a consistent trend of maintaining a β-sheet secondary structure. Amylin plus SK9 at 32 μM (C) shows nearly no change in the secondary structure over a 24 h period as the spectra seem to only increase in intensity at the β-sheet negative band at 218 nm.

To corroborate the results of ThT assays, circular dichroism (CD) spectroscopy was employed to monitor conformational transitions of amylin in the presence and absence of SK9 (Figure 8). Changes observed in the spectra of the amylin control are consistent with a secondary structure shift from random coil/partial α helix (negative band at 205 nm at 0 h) to β sheet (positive band at 196 nm and a negative band at 218 nm).^37−39^ This β-sheet conformation was found to persist up to 4 days, at which point data collection was ceased. The spectra of amylin incubated with SK9 in a 1:1 ratio display slight structural changes during the first 3 h and indicate some initial helical content, evidenced by the minimum at approximately 208 nm at time 0. However, after 3 h, the spectra consistently show the presence of a β-sheet secondary structure. Subtle differences can be seen when amylin is incubated with SK9 in a 1:4 ratio. Under these conditions, no major change in the secondary structure can be seen from time 0 onward. The spectra only increase in intensity of the negative band at 218 nm, indicating a growing level of the β-sheet structure. In contrast to the amylin control, the lack of initial helical content in amylin with a higher SK9 concentration implies that the viral peptide may actually hasten β-sheet formation. Ultimately, circular dichroism studies demonstrate that when amylin is incubated with SK9 in either a 1:1 or 1:4 ratio, amylin still assembles into β-sheet structures consistent with amyloid fibrils. These findings support our ThT data and again provide evidence that SK9 does not stabilize amylin in its native conformation and slows or prevents its self-assembly. Careful examination of the CD data implies that SK9 may somewhat enhance amylin aggregation, although this enhancement was not detectable in the ThT assay. Regardless, clearly, SK9 has a minimal effect in preventing amylin aggregation over the concentration range examined. Finally, CD spectra of SK9 alone confirmed that this peptide is incapable of forming amyloid, as it persists in a random coil conformation throughout the time course (Figure S2).

**Figure 9:**
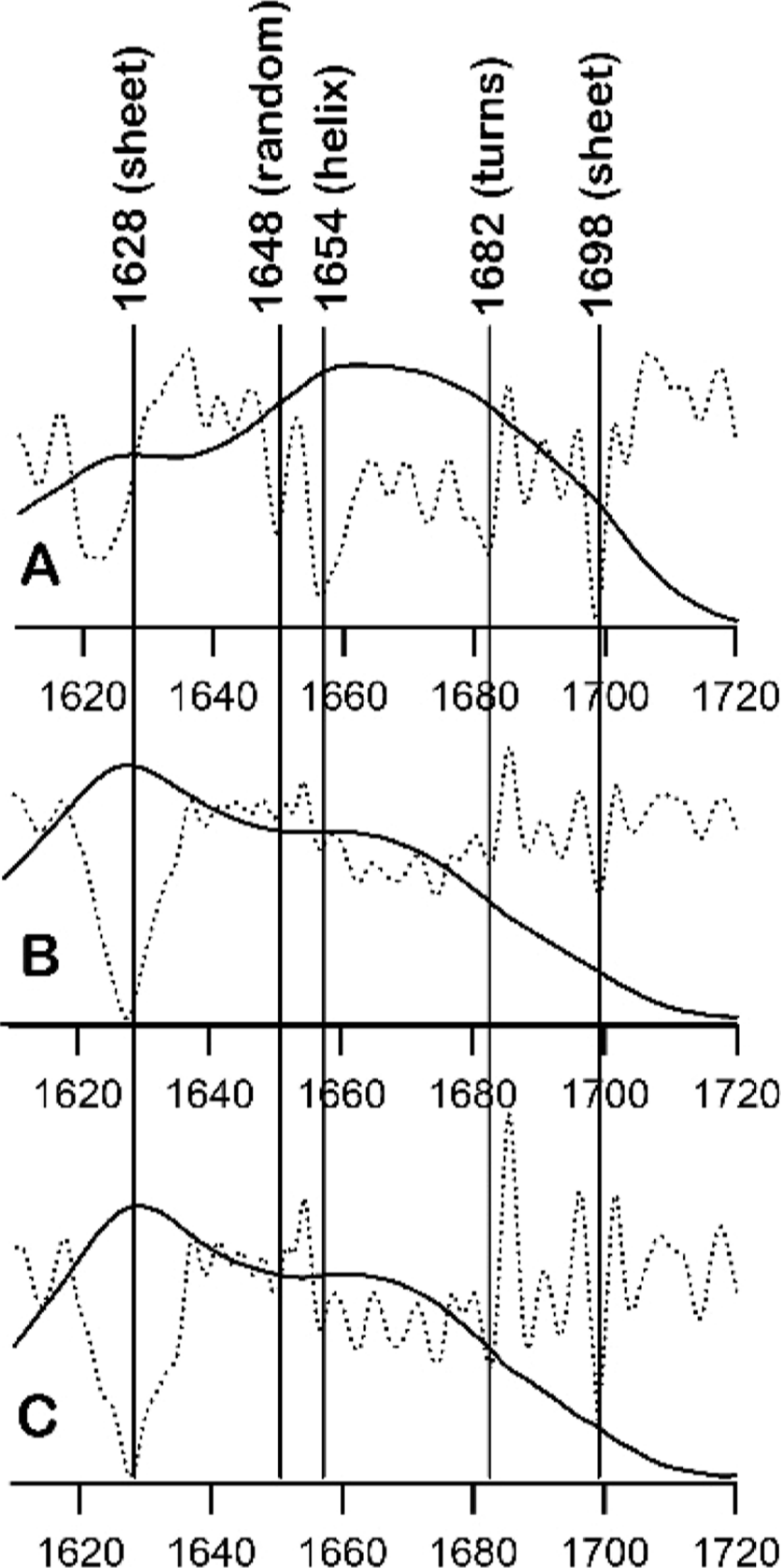
FTIR spectra (solid lines) and its second derivate (broken lines) to accentuate the peaks of the amide I region of the initial aliquot of amylin in water (A) and washed aggregates collected from amylin (B) alone and amylin in the presence of SK9 (C).

We next examined the Fourier transform infrared (FTIR) spectra of aggregates formed by amylin in the absence and presence of SK9. Proteins exhibit several distinct amide vibrational frequencies, ranging from 3300 to 200 cm^–1^.^40^ The most commonly used amide regions in FTIR studies of amyloid fibrils are amide I (1600–1700 cm-1),^41−43^ amide II (1520–1540 cm^–1^),^44^ and amide III (1220–1240 cm^–1^).^45−47^ Among these, the amide I region is the most sensitive to the polypeptide backbone secondary structure and the least affected by side chain vibrations.^48^Figure 9 shows the FTIR spectra, along with the double derivative to accentuate peak positions of the amide I region, of dried amylin samples. The initial aliquot of amylin displayed an intense peak between 1650 and 1660 cm^–1^, indicative of α-helical conformations^49^ (Figure 9A) consistent with the solution NMR structure of human amylin.^21^ The same sample also revealed peaks at ca. 1648 cm^–1^ (random coils) and 1682 cm^–1^ (turns).^50^ Interestingly, a relatively broader peak at ca. 1621 cm^–1^ and a weaker band at ca. 1679 cm^–1^ were both observed in the established regions for amyloid β sheet (Figure 9A).^43,50^ It is possible that a fraction of amylin had already started to aggregate and that the broadening of the 1621 cm^–1^ peak was due to the presence of various β conformations during the structural transition. As expected, the spectrum of dried amylin aggregates lacked intense α-helix and random coil peaks (Figure 9B). Only bands for β sheet, a sharp peak at ca. 1629 cm^–1^ and a weaker peak at ca. 1698 cm^–1^, and β turn, peaks within the 1660–1682 cm^–1^ region,^50,51^ were observed which is consistent with previous FTIR-based studies of amyloid fibrils.^42,52^ Interestingly, incubation with SK9 appeared to exhibit little to no effect on the FTIR bands of amylin aggregates, further solidifying our previous results, suggesting that amylin aggregation is not affected by the presence of SK9.

**Figure 10:**
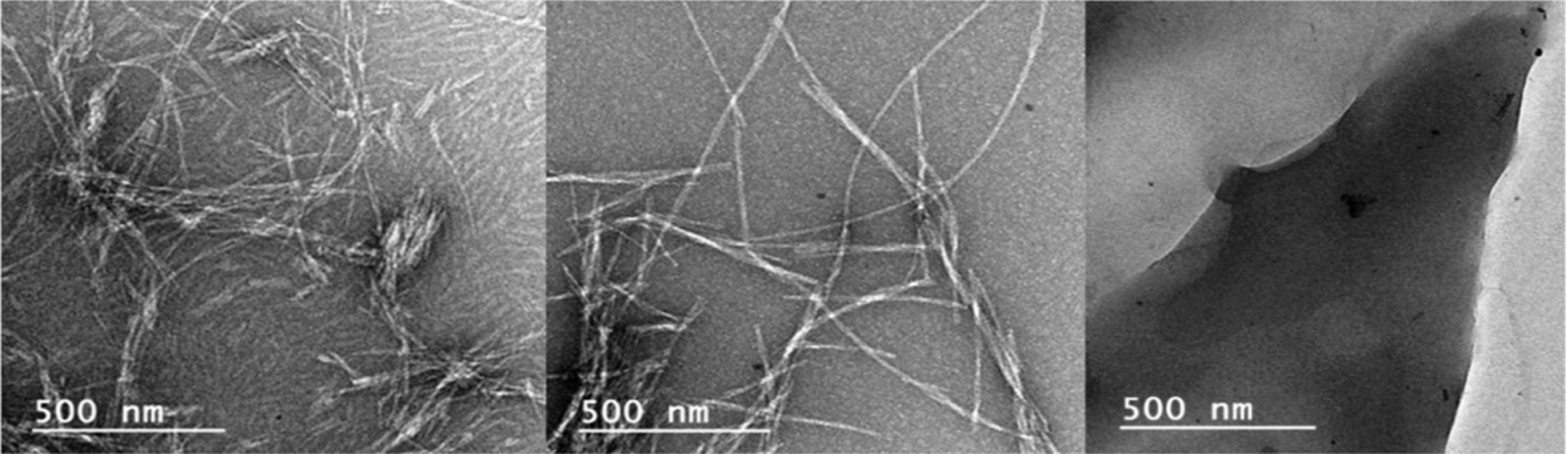
TEM images of amylin alone (left panel), amylin incubated with SK9 (middle panel), and SK9 alone (right panel). Images were taken of aged CD samples deposited on Formvar carbon-supported nickel grids stained with 2% uranyl acetate. The first two images showed the formation of fibrils, whereas SK9 alone on the far right did not. Amylin incubated with SK9 showed a similar morphology but finer fibers compared to amylin alone. The presence of fibers for amylin alone or amylin incubated with SK9 and the lack of fibril formation for SK9 alone validate both the ThT and CD data collected.

Finally, transmission electron microscopy (TEM) confirmed the physical presence of amyloid and documented the fibril morphology (Figure 10). From the electron micrographs, it is evident that amyloid fibrils are present in the amylin control as well as the sample incubated with an equimolar concentration of SK9. These findings again corroborate ThT, CD, and FTIR data and provide further proof that SK9 does not prevent aggregation of full-length amylin. Likewise, TEM images also verify the inability of SK9 to form amyloid.

Multiple MD studies have alluded to potential pathological interactions between SARS-CoV-2 proteins or protein fragments and amyloidogenic proteins.^22,23^ Indeed, the link between SARS-CoV-2 infection and specific disease-associated amyloidogenic proteins has been documented. SARS-CoV-2 proteins have been shown to bind serum amyloid A and α synuclein, resulting in their upregulated expression and thus elevated amyloid formation.^53,54^ Additionally, simulations by Coppola et al.^55^ have demonstrated that amyloid-β fibrils can interact with the receptor binding domain of the viral spike protein, suggesting a potential mechanism through which SARS-CoV-2 proteins may exacerbate or interfere with neurodegenerative disorders. In this study, we demonstrated that a peptide fragment of the SARS-CoV-2 envelope protein can inhibit the formation of amyloid by hIAPP_12–18_ and hIAPP_20–29_. Of the two hIAPP peptides, SK9 has the greatest effect on hIAPP_12–18_. SK9 abolishes the ability of LANFLVH to form aggregates. Our docking and MD simulations provide some insight into the apparent differences in the ability of SK9 to interact with the two sequences. Aromatic π stacking appears to be a central feature of the interactions between SK9 and the amylin fragments. In the case of hIAPP_20–29_, Phe-23 and Tyr-59 participate in a single aromatic ring stacking interaction. In contrast, hIAPP_12–18_ and SK9 form two sets of aromatic–aromatic contacts. These involve Phe-15 and His-18 which pair with Tyr-59 and Phe-56 of SK9, respectively. Consistent with this, HADDOCK docking studies provide an average free energy of binding for the SK9/hIAPP_20–29_ complex of −78 kJ/mol, whereas the SK9/hIAPP_12–18_ complex displays an average binding energy of −101 kJ/mol. The higher affinity of SK9 for the hIAPP_12–18_ sequence likely contributes significantly to the viral peptides’ ability to better inhibit aggregation. In the case of hIAPP_20–29_, the affinity of SK9 for this sequence may be insufficient to prevent self-assembly, resulting in a lower overall yield of amyloid.

Another interesting feature that distinguishes the binding modes of SK9 to the amylin sequences is that binding to hIAPP_20–29_ occurs in an antiparallel orientation, while binding to hIAPP_12–18_ proceeds in a parallel fashion.

Since SK9 displays a greater effect on hIAPP_12–18_ than hIAPP_20–29_, we posit that this is its primary region of interaction with full-length amylin. However, while this interaction is strong enough to inhibit the aggregation of hIAPP_12–18_, it is not enough to disrupt amyloid formation by full-length amylin. This may be due to several reasons. If one considers that the “amyloidogenic core” consisting of residues 20–29 does not interact strongly with SK9, then this region is likely still available in the native polypeptide to drive self-assembly. In addition, while the 20–29 region is often termed the amyloidogenic core, the fact remains that there are several stretches of amyloidogenic regions in amylin, including the C-terminal region. Disruption of a single amyloidogenic region by SK9 is likely not sufficient to overcome the cumulative effect of the other remaining amyloidogenic sequences. Furthermore, while recent work has emphasized the often-unrecognized contribution of the hIAPP_12–18_ sequence to amylin self-assembly,^25^ it still stands to reason that disruption of this single region may not be enough to disrupt the aggregation process.

The development of type 2 diabetes has been associated with COVID-19 and may be a long-term complication of the disease. It has been speculated that COVID-19, in conjunction with preexisting metabolic syndrome and associated islet remodeling, may accelerate development of type 2 diabetes.^13^ While several potential mechanistic pathways exist that may be responsible for the onset of type 2 diabetes in the setting of COVID-19, some have been postulated to involve full-length hIAPP. These hypotheses include dysfunction and disruption of the lysosomal system in which misfolded hIAPP is not destroyed, resulting in aggregation and amyloid deposition. Inflammatory cytokines such as IL-1β, IL-6, and TNF-α that are characteristic of the COVID-19 “cytokine storm” may also contribute to ongoing or preexisting islet cell inflammation and remodeling, culminating in the loss of β-cell mass.^13^ Another hypothesis is that SARS-CoV-2 proteins may directly interact with hIAPP and alter its amyloidogenic potential. MD simulations by Chesney et al.^20^ indicate that SK9 does not make hIAPP more prone to aggregation but actually stabilizes its native conformation. This implies that SK9 could serve as a potential amylin aggregation inhibitor. In our experiments, however, we did not observe any significant acceleration or inhibition of amyloid formation by hIAPP in the presence of SK9. These findings strongly suggest that SK9 does not directly interact with amylin in a manner that affects its aggregation.

Chesney and co-workers,^20^ through their simulations, also observed that SK9 can stabilize hIAPP fibrils and posit that such stabilization may shift the equilibrium toward toxic amyloids, thereby increasing the risk for type 2 diabetes. Our experimental data show no significant increase in the total yield of amyloid produced when hIAPP is incubated with SK9 compared to the amylin control. Thus, we conclude that SK9 has little to no influence on the self-assembly kinetics of full-length amylin.

## Conclusions

Using MD simulations and biophysical techniques, we have demonstrated that SK9, a nine-residue fragment of the SARS-CoV-2 envelope protein, can interact with short peptides derived from hIAPP. SK9 is capable of interacting with both hIAPP_12–18_ and hIAPP_20–29_ inhibiting their ability to self-assemble. Notably, SK9 displays selectivity between the two amylin sequences and interacts more strongly with hIAPP_12–18_, virtually abolishing its ability to form amyloid. Furthermore, we provide direct experimental evidence that despite the ability of SK9 to prevent the aggregation of short hIAPP sequences, the viral peptide is incapable of blocking amyloid formation by full-length amylin. These observations contradict previous MD studies, suggesting that SK9 could stabilize amylin in its native conformation and prevent its misfolding and self-assembly. By dispelling the possibility of direct binding of the viral envelope protein or its fragments to full-length amylin, the experimental data presented in this study may help narrow the spectrum of potential mechanisms by which SARS-CoV-2 infection can induce type 2 diabetes.

## Materials and Methods

Standard 9-fluorenylmethyloxycarbonyl (Fmoc)-protected amino acids, Rink amide resin, dimethylformamide (DMF), and all other reagents for peptide synthesis were purchased from Reagents Holdings (Louisville, KY) unless otherwise noted. Dichloromethane and acetonitrile were obtained from Thermo Fisher Scientific (Waltham, MA). Amylin was purchased from Peptide 2.0 (Chantilly, VA) or Bachem (Torrance, CA). All other reagents were from Sigma-Aldrich (St. Louis, MO). Copper- and nickel-coated Formvar grids for TEM were purchased from Electron Microscopy Sciences (Hatfield, PA).

### MD and Docking Simulations

MD simulations were used to investigate the effect of SK9 on the structural stability of amylin fragments hIAPP_12–18_ and hIAPP_20–29_. Since the C-terminal tail of the envelope protein of SARS-CoV-2 is not yet resolved, we obtained the structure of SK9 generated by Jana et al.^22^ using machine-learning methods and successive refinement.^56^ The initial configurations of hIAPP_12–18_ and hIAPP_20–29_ were extracted from the NMR-resolved structure of hIAPP (PDB ID: 2L86).^21^ Each amylin fragment was docked with SK9 in a 1:1 ratio using HADDOCK.^30,31^ The configuration with the best HADDOCK score was selected as the starting point for the MD simulation.

The systems for MD simulation, i.e., the fragments alone and docked with SK9, were each placed in a cubic box filled with TIP3P^57^ water molecules where the edges are distanced 1.5 nm from the sequences. Simulations were run using the GROMACS 2022 package^32^ with the CHARMM 36m all-atom force field.^33^ Energy minimization was done using position restraints and terminated upon fulfillment of the energy criteria (maximum force <1000 kJ/mol/nm). This was followed by equilibration, first with constant number of particles, volume, and temperature for 100 ps and then with constant pressure instead of volume for another 100 ps. Position restraints were also employed in both equilibration processes. After equilibration, MD simulations were performed for each system at a constant temperature and pressure of 300 K and 1 atm, respectively, with no position restraints. This means that the sequences can move and detach during the simulation.

Electrostatic interactions were calculated via the particle-mesh Ewald method.^58^ Temperature and pressure were regulated using a v-rescale thermostat^59^ and Parrinello–Rahman barostat,^60^ respectively. Water geometry and nonwater bonds including hydrogen atoms were constrained with SETTLE^61^ and LINCS^62^ algorithms, respectively. The time step was set to 2 fs, with data being stored every 2 ps.

The Dictionary of Secondary Structure in Proteins (DSSP)^63^ algorithm was utilized to assess the secondary structure trajectory of the systems. The rmsd with respect to the initial configuration was obtained using the GROMACS gmx_rms tool. PYMOL software was used for visualization.^64^

### Peptide Synthesis

Peptides were synthesized manually on Rink amide resin (substitution level = 0.3 mmol/g) using a standard Fmoc solid phase synthesis protocol. Briefly, Fmoc removal was accomplished by using 20% piperidine (v/v) in DMF for 20 min. Couplings were performed using 3 equiv each of Fmoc-protected amino acid, *N,N,N′,N*′-tetramethyl-*O*-(1H-benzo-triazol-1-yl)uranium hexafluorophosphate, and 1-hydroxy-benzotriazole and 9 equiv of *N*-methylmorpholine relative to resin-bound amine. Amino acid couplings and Fmoc removal were monitored by the Kaiser ninhydrin test.^65^ Peptides were cleaved from the resin with a cocktail of 95% trifluoracetic acid, 2.5% water, and 2.5% triisopropylsilane (TFA/H_2_O/TIPS) for 2 h at room temperature. Crude peptides were precipitated and washed with cold diethyl ether. Crude peptides were dissolved in 50% acetonitrile/water containing 0.1% TFA, filtered through a 0.45 μM PVDF membrane, and purified by reverse-phase high pressure liquid chromatography (HPLC) (Varian ProStar, Palo Alto, CA) on a Vydac C_18_ protein and peptide column (2.2 × 25 cm) with a linear gradient of CH_3_CN/H_2_O containing 0.1% TFA. Column effluent was monitored at 218 and 254 nm. Appropriate fractions were pooled, concentrated, and lyophilized to provide peptides as a fluffy white solid. The peptide structure was confirmed by matrix-assisted laser desorption ionization time-of-flight mass spectrometry (Waters Corporation, Milford, MA). Peptide purity was assessed by reverse-phase analytical HPLC on a Vydac C_18_ protein and peptide column (4.6 × 250 mm).

### ThT Fluorescence Assays

Assays were conducted in 96-well format using black-walled, nonbinding, microtiter plates (PerkinElmer, Shelton CT). Lyophilized hIAPP_12–18_, hIAPP_20–29_, and SK9 peptides were each dissolved in dimethyl sulfoxide (DMSO) to prepare concentrated stock solutions. Stock solutions were sonicated immediately prior to use, and aliquots were diluted into 10 mM Tris-HCl buffer (pH 7.4) containing ThT with final peptide and ThT concentrations of 200 and 10 μM, respectively. To this mixture, SK9 was added to a final concentration of 200 μM. Measurements were performed on a VICTOR^3^ V 1420 Multilabel Counter (PerkinElmer, Shelton, CT). Samples were excited at 450 nm, and fluorescence emission was monitored at 486 nm. Fluorescence intensity was measured every 15 min for at least 20 h at room temperature. For assays utilizing full-length amylin, aliquots of concentrated stocks of SK9 in DMSO were added to 10 mM Tris-HCl buffer (pH 7.4) containing ThT followed by amylin in DMSO to provide solutions containing final concentrations of 20, 40, and 80 μM SK9, 20 μM amylin, and 4 μM ThT. The spectral response from each sample well was measured every 15 min for 24 h as previously described. Assay data were obtained in triplicate and fitted to a four-parameter sigmoidal curve using Igor software.

ThT fluorescence spectra of SK9 were acquired on a Fluoromax-4 spectrofluorometer (Horiba Jobin Yvon Inc., Edison, NJ) with a 1 cm quartz cuvette at 25 °C using an excitation wavelength of 450 nm and slit of 2.5 nm. The emission slit was set to 10 nm starting at 450 nm and ending at 510 nm. Samples were prepared by adding SK9 at varied concentrations (20 and 40 μM, respectively) to a buffer solution (10 mM Tris-HCl, 100 mM NaCl, 0.02% NaN_3_, pH 7.5) containing ThT (20 μM). The capped cuvette was inverted slowly to mix the solution before collection.

### Circular Dichroism

CD spectra were collected as a function of time to monitor secondary structure changes of amylin in the absence and presence of SK9. Lyophilized peptides were dissolved in 100% 1,1,1,3,3,3-hexafluoroisopropanol to prepare concentrated stock solutions. Stock solutions were sonicated for 30 min directly prior to use. Aliquots of concentrated stocks were diluted into 10 mM Tris-HCl buffer at a pH of 7.5 in a 1 cm path length quartz fluorometer cuvette. To this mixture, amylin was added to a final concentration of 8 μM. The capped cuvette was inverted slowly to mix the solution before measuring the spectra using a Jasco J-810 spectropolarimeter (Jasco Inc., Easton, MD) for at least 24 h. Measured samples included an amylin control at 8 μM, SK9 alone at 8 μM, SK9 alone at 32 μM, amylin plus SK9 at a 1:1 ratio at 8 μM, and amylin plus SK9 at a 1:4 ratio at 8 and 32 μM, respectively. Spectra were baseline-corrected by subtracting the spectra of buffer alone from the spectra collected for amylin and SK9 alone. The amylin–SK9 mixtures were corrected by subtracting the spectrum of the solution containing SK9 in a buffer for each respective concentration.

### FTIR Characterization of Peptide Aggregates

Samples for FTIR measurements were prepared by incubating 1 mM amylin alone or with 1 mM SK9 in water at room temperature. To probe the initial characteristics of amylin, an aliquot of the freshly prepared amylin solution was immediately applied to a single AgCl cell and dried in a desiccator. After 24 h of incubation, the samples were centrifuged to remove the supernatant, while the resulting aggregates were washed three times with distilled water. The aggregates were then applied to a AgCl cell and allowed to dry. The prepared samples were analyzed using a Nicolet iS50 FTIR spectrometer with a sample compartment prepurged with CO_2_-free dry air and a liquid nitrogen-cooled mercury–cadmium–tellurium detector (Thermo Scientific, Madison, WI). Final FTIR spectra were an average of 150 scans collected at a resolution of 4 cm^–1^. To better identify peak positions in each amide region, a second derivative of each spectrum was taken by using Omnic 9.13 FTIR software (Thermo Scientific, Madison, WI).

### Transmission Electron Microscopy

To visualize the aggregates of amylin alone, amylin incubated with SK9, and SK9 alone, TEM images were collected using aged assay solutions. Each grate was prepared by depositing 7 μL of sample onto a 300 mesh Formvar carbon-supported nickel grid. After the sample was left to dry for several minutes, the grid was then stained with 7 μL of 2% uranyl acetate and instantly blotted to avoid overstaining. Images were captured using a JEOL JEM2100 multipurpose (LaB6) 200 kV electron microscope (Jeol USA, Inc., Peabody, MA) equipped with two digital cameras: a Gatan Orius 833 slow scan charge-coupled device (CCD) camera (2048 × 2048 pixels) for large field of view and a Gatan Ultrascan 1000XP CCD camera (2048 × 2048 pixels, 14bit) for high-resolution imaging.

## Supporting Information Available

ThT fluorescence spectra of freshly dissolved SK9 after 16 days of incubation and time course CD spectra of SK9 alone (PDF)

## Abbreviations

hIAPP: human islet amyloid polypeptide
SARS-CoV-2: severe acute respiratory syndrome coronavirus 2
SK9 sequence: SFYVYSRVK
hIAPP_12–18_ sequence: LANFLVH
hIAPP_20–29_ sequence: SNNFGAILSS
MD: molecular dynamics
DSSP: Dictionary of Secondary Structure in Proteins
rmsd: root mean square deviation
ThT: thioflavin T
CD: circular dichroism
CCD: charge-coupled device
TEM: transmission electron microscopy
FTIR: Fourier transform infrared
Fmoc: 9-fluorenylmethyloxycarbonyl
TFA: trifluoroacetic acid
DMSO: dimethyl sulfoxide
TIS: triisopropylsilane
