# Supplementary material for: Effect of a SARS-CoV-2 Protein Fragment on the Amyloidogenic Propensity of Human Islet Amyloid Polypeptide
Source: ACS Chem Neurosci. 2024 Nov 25;15(24):4431–40. doi: 10.1021/acschemneuro.4c00473 (PMC11660541; doi:10.1021/acschemneuro.4c00473)
Supplement: Supplementary file 1 — cn4c00473_si_001.pdf [file cn4c00473_si_001.pdf]

Effect of a SARS-CoV-2 Protein Fragment on the Amyloidogenic Propensity of Human Islet Amyloid Polypeptide

Marvin Bilog,[†,‡] Jennifer Cersosimo,[†,‡] Iliana Vigil,[‡] Ruel Z. B. Desamero[†,‡,] and Adam A. Profit[†,‡,*]*

[†]PhD Programs in Chemistry and Biochemistry, The Graduate Center of the City University of New York, New York, New York 10016, USA

[‡]Department of Chemistry, York College of the City University of New York, Jamaica, New York, 11451, USA

*Corresponding authors:

Email address: rdesamero@york.cuny.edu for RZBD and aprofit@york.cuny.edu for AAP

Supplementary Information

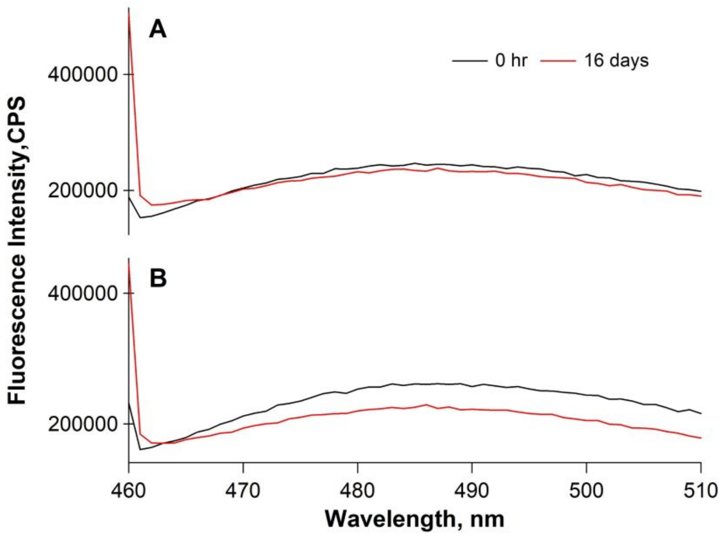

Figure S1. ThT fluorescence spectra of freshly dissolved SK9 at 20 μM (A) and 40 μM (B) at time zero (black) and after 16 days of incubation (red). After 16 days, SK9 did not exhibit any significant increase in fluorescence intensity.

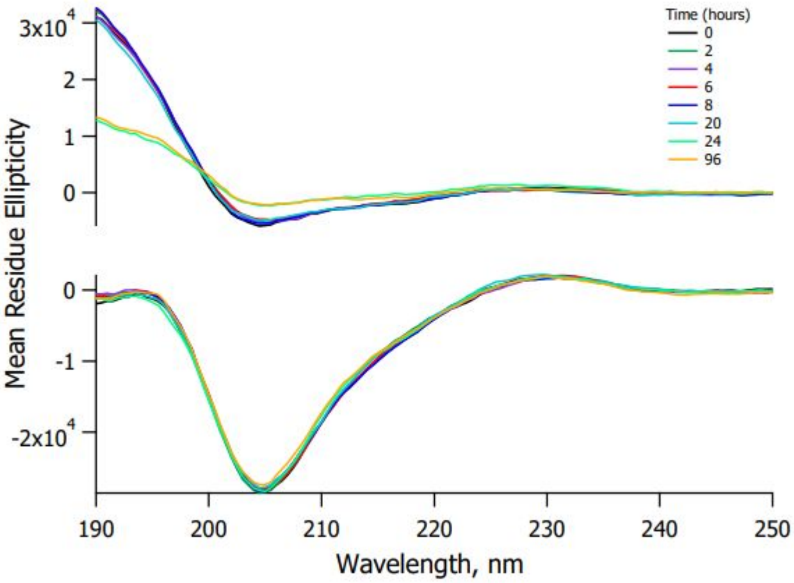

Figure S2. Time course of SK9 alone as monitored by CD. SK9 alone at 8 μM (top) shows partial helical character which seems to maintain its structure over a 23-hour period. SK9 alone at 32 μM (bottom) showed a similar CD profile to SK9 alone at 8 μM but with a larger intensity. The lack of negative band at 222 nm, and the 208 nm band moving towards a lower wavelength reflects increasing amounts of random coil.
